# Studying GPCR/cAMP pharmacology from the perspective of cellular structure

**DOI:** 10.3389/fphar.2015.00148

**Published:** 2015-07-17

**Authors:** Peter T. Wright, Sophie Schobesberger, Julia Gorelik

**Affiliations:** Functional Microscopy, Myocardial Function, National Heart and Lung Institute, Imperial College London, Du Cane Road, London, UK

**Keywords:** GPCRs, cAMP, compartmentation, caveolae, T-tubules, lipid rafts, scanning ion conductance microscopy, FRET sensors

## Abstract

Signal transduction via G-protein coupled receptors (GPCRs) relies upon the production of cAMP and other signaling cascades. A given receptor and agonist pair, produce multiple effects upon cellular physiology which can be opposite in different cell types. The production of variable cellular effects via the signaling of the same GPCR in different cell types is a result of signal organization in space and time (compartmentation). This organization is usually based upon the physical and chemical properties of the membranes in which the GPCRs reside and the repertoire of downstream effectors and co-factors that are available at that location. In this review we explore mechanisms of GPCR signal compartmentation and broadly review the state-of-the-art methodologies which can be utilized to study them. We provide a clear rationale for a “localized” approach to the study of the pharmacology and physiology of GPCRs and particularly the secondary messenger cAMP.

## Introduction

The members of the G-protein coupled receptor (GPCR) family act through multiple pathways upon activation as they possess the ability to bind a panel of G-proteins and β-arrestins. As a result ligand binding can potentially activate multiple effector pathways with differential effects upon cellular physiology. The array of these effectors is vast and outside of the scope of both this article and this review series, readers are therefore encouraged to consult previous reviews (Neves et al., 2002; Kenakin, 2011; Shukla et al., 2011). However, in the setting of specialized tissues and cells GPCRs are located into specific compartments with defined molecular profiles, which strictly determine the potential physiological outcomes of signaling. The consequence is that although GPCR signaling is potentially “omnidirectional,” in reality signaling outcomes are restricted by the accessibility of secondary signaling molecules such as cyclic adenosine monophosphate (cAMP) and cyclic guanosine monophosphate (cGMP) and the presence of their modulators such as phosphodiesterases or protein kinases activated by the cyclic nucleotides. For a comprehensive and exhaustive review on the cyclic nucleotides and their modulators in cardiac cells the reader is encouraged to refer to the works of the Zaccolo and Movsesian (2007) or the Conti group (Conti and Beavo, 2007).

**FIGURE 1:**
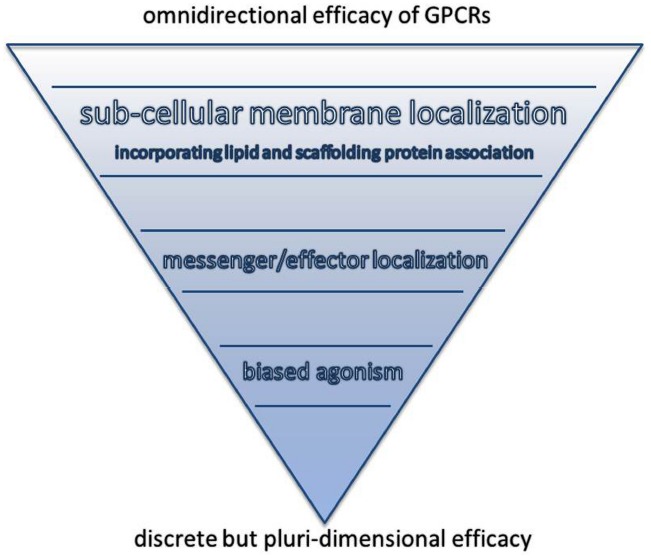
**Schematic representation of the mechanisms shaping the control of GPCR signaling.** The efficacy of a ligand binding to a GPCR is potentially omnidirectional. Meaning that in theory any physiological outcome can be produced following the signal transduction event. However, through various levels of intermolecular and, ultimately, cellular organization the efficacies of ligands and GPCR function become essentially discrete.

In addition to the diversity of signaling partners, the GPCRs’ status as transmembrane proteins ensures that local plasma membrane properties perform a central role in the production of downstream signaling effects. If we set aside differences in the expression profiles of specific GPCRs, three major mechanisms contribute to shape GPCR signaling; these are the biased agonism of GPCRs (Violin et al., 2014), secondary messenger compartmentation (Perera and Nikolaev, 2013) and modulation by lipid raft association (Escribá et al., 2007). All of these phenomena will be discussed later in this review (See Figure 1).

Over the last decades numerous methodological and technological advances have enabled researchers to better determine the localized pharmacology of GPCRs from the perspective of cell membrane structure. However, the advancement of methods for studying the mechanisms of local modulation of GPCR signaling to ever greater resolution remains a necessity. Equally, deeper investigation of the central organizing principles of this signaling compartmentation at a subcellular level is required. These advances will allow a better understanding of the ways in which GPCRs shape cellular physiology. There is a desire to create drugs based upon improved ligands (Shonberg et al., 2014) for GPCRs producing very specific (ultra-biased) mono-directional signaling to reduce off-target effects. This will only be realized after rigorous assessment of the ways in which, sub-cellular micro-domains modulate GPCR signaling, in health and within the context of pathologies. This current is gaining momentum, particularly within the cardiovascular field; cardiac muscle has been shown to display examples of highly defined GPCR signal regulation. In this paper the pharmacology of multiple GPCRs will be discussed but the adrenoceptors (ARs) are frequently used as examples, due to them being seen as a prototypical GPCR by many researchers. This has led to a large amount of work being focused upon their physiology and pharmacology. They are also of special interest to the authors.

## The Concept of the Structural GPCR Microdomain

The effect of a specific ligand on a GPCR is in theory, identical in all tissues, however, these signaling events often produce different effects in different cells. For example the binding of an agonist such as adrenaline to a βAR will increase the contractility of cardiac muscle but reduce the contractility of airway smooth muscle (Brodde, 1993; Barnes, 1995). To allow for cell type-specific divergence, in the downstream effectors of signaling must be locally organized in space and time (Bethani et al., 2010). This allows a single stimulus to result in a biologically relevant whole organ/organism response. The difference mentioned above is due to the altered targeting of protein kinase A-regulatory type 2 (PKA-RII) by the secondary messenger cAMP within the two different cell types. In cardiac muscle PKA phosphorylates L-Type Ca^2+^ channels (LTCC) and phospholamban (PLB), the effect of which is to increase the amplitude and speed of the cellular Ca^2+^ transient (Brodde, 1993). In smooth muscle cells an increase in cAMP activates PKA but with the effect that the myosin light chain kinase (MLCK) is phosphorylated thereby reducing its affinity for the Ca^2+^/calmodulin complex and lowering contractility (Barnes, 1995). Glucagon receptors (GLU-R) activate glycogen phosphorylase and cause positively inotropic and lusitropic effects in cardiomyocytes (Farah, 1983). G_*s*_-linked receptors like GLU-R may cause divergent effects, for example Glucagon-like peptide-1 receptors (GLP1R) cause negatively inotropic effects (Vila Petroff et al., 2001). β_2_AR is also G_*s*_-linked but its effects upon cardiomyocyte inotropy are somewhat equivocal. Some studies report it to exert no effect upon relaxation and it cannot activate glycogen phosphorylase like Glu-R (Xiao and Lakatta, 1993; Kuznetsov et al., 1995). However, other studies report changes in inotropy (Bartel et al., 2003). We discovered that β_2_AR stimulation affected inotropy to a different degree depending on which region of the heart the cells were isolated from (Paur et al., 2012). Equally, we discovered that the cAMP responses of G_*s*_-linked β_1_AR and β_2_AR were quantitatively different at the sub-cellular level (Nikolaev et al., 2010). In these cases different receptors are signaling via the same G-protein and the outcome is the same in terms of signal transduction, i.e., increases in cAMP, but due to differing cellular organizations the signal is interpreted differently and the contractile outcome is divergent.

### Cyclic Nucleotide Compartmentation

A large and increasing body of work has described biological mechanisms which serve to shape intracellular cAMP pools (Mika et al., 2012; Perera and Nikolaev, 2013; Guellich et al., 2014). This secondary messenger is produced upon the activation of adenylate cyclase (AC) following the dissociation of the stimulatory-G (G_*s*_) protein from specific classes of GPCRs. Compartmentation of cAMP regulatory mechanisms appears to be a structural phenomenon specific to cell type. This area of study began to accrete following the pioneering work of Buxton and Brunton (1983) who asked how it was that two agents (Prostaglandins and Isoprenaline), which serve to increase cellular cAMP concentrations via different receptor pathways produce differing effects upon cellular physiology.

cAMP produces its effects within cells by causing the activation of PKA (Zaccolo, 2009), exchange protein activated by cAMP (Epac; Métrich et al., 2008) or cyclic nucleotide gated channels (CNGCs; Rochais et al., 2004). Furthermore the cAMP signaling domains are at once both physical and virtual compartments. They rely upon the formation of molecular networks which involve the close apposition of plasma membrane and cellular organelles such as sarcoplasmic reticulum; in addition, the sub-cellular localization of phosphodiesterases, phosphatases and tertiary effectors as well as important protein associations. The central principle of cAMP compartmentation is that cAMP must be present in the vicinity of cAMP-dependent effectors (PKA, Epac, or CNGCs) to cause the transduction of signaling into physiological changes within the cell (Di Benedetto et al., 2008). However, it must be prevented from diffusing from the effector compartment. Therefore cAMP is either degraded or actively extruded from cells through an energy consuming ATP anion pump (Wiemer et al., 1982). Stringent control of cAMP levels assures that a discrete panel of effectors is being activated as a result of a particular signaling event. The differential activities of Prostaglandin and Isoprenaline are due to the activation of different pools of PKA within the cardiomyocyte. Due to stringent control of its localization, cAMP produced as a result of the activation of Prostaglandin receptors cannot cause the cAMP-dependent PKA mediated phosphorylation of members of the excitation-contraction coupling pathway within cardiomyocytes in the manner of the AR.

Cells organize their effector molecules on the basis of the specific needs, and as a result the efficacy of a given agonist in a cell type effectively becomes discrete. Consequently gross changes in cellular structure or gene expression in response to pathology which involve this secondary messenger physiology must always be viewed through this prism. Due to the fact that physiological effects represent the sum of many stringently controlled local events it is of great importance to study localized cell signaling.

### Membrane Organization

Cyclic AMP compartmentation is heavily controlled by membrane structures. The following paragraphs will illustrate the involvement of membranous subcellular domains in the regulation of signaling compartmentation.

#### Lipid Rafts

The structure of the lipid bi-layer is essential for maintaining GPCRs’ defined molecular structure, and therefore, their function. The lipid make-up of the membrane is not homogeneous. Indeed, for the past three decades researchers have been aware of detergent insoluble components of the lipid bilayer (Simons and Ikonen, 1997). Many fundamental studies have been carried out to establish the effect of membrane composition upon GPCR function. Many of these have utilized rhodopsin due to this molecules status as an archetypal GPCR for structural studies (Botelho et al., 2002). It appears that a flexible membrane composed of a greater amount of lipids with phosphatidylethanolamine (PE) head groups and docosahexaenoyl chains (DHA), shift rhodopsin toward its active state. The presence of greater quantities of cholesterol increases the rigidity of membranes and as a result has been demonstrated to drive rhodopsin toward its inactive state. The same study demonstrated this was also true for the depletion of DHA (Feller and Gawrisch, 2005). The Singer and Nicolson (1972) fluid mosaic model was postulated and states that the cell membrane is a fluid bilayer through which protein constituents are able to float freely. This “lamellar” structure has been observed to be a basic state of the cell membrane upon which more complex states are superimposed. The basic state described above is defined as being liquid crystalline (Yeagle, 2004). Other states observed are known as gel, pseudo-crystalline, rippled and liquid ordered (Brown and London, 1997). The liquid ordered phase is the most interesting of these states from the perspective of GPCR biology. This configuration is also frequently described as being a “lipid raft,” as these phases represent sub-regions of the liquid membrane (Simons and Vaz, 2004). These structures are produced by concentrating the acyl chains of lipids which were in a gel phase. The result of which is the preservation of a degree of lateral mobility. Lipid rafts are enriched in cholesterol and glycosphingolipid and represent about 30% of cellular membranes. They appear to be intrinsically important in modulating GPCR function (Oates and Watts, 2011). Scaffolding molecules such as the A-kinase anchoring proteins (AKAPs), which organize PKA effectors and stabilize the interaction of phosphodiesterases within their domains, are also thought to be localized to lipid rafts where they cluster with the effectors (Kritzer et al., 2012). Caveolae are by far the most well studied lipid raft domains within the context of GPCR and cAMP signaling. The role of these domains in controlling GPCR function is detailed below.

#### Caveolae

Caveolae are specialized lipid raft domains found in the plasma membrane of many cell types. They are classed as a distinct type of lipid raft as they contain specialized scaffolding proteins such as caveolins and cavins. In two-dimensional transmission electron microscopy (TEM) studies caveolae appear as 50–100 nm in diameter flask-shaped regions of the lipid bilayer. Thus in the three dimensional environment of the cell membrane they are bulb-like invaginations with restricted mouths open to the extracellular environment (Razani et al., 2002). They appear to be formed via the concentration of cholesterol and the aforementioned scaffolding peptides. Caveolae are responsible for cell signaling, lipid storage and endocytosis. The majority of cellular studies have historically relied on TEM to visualize these domains and the cholesterol chelating agent methyl-β-cyclodextrin (MβCD) to disrupt them (Harvey and Calaghan, 2012). As a pharmacological or physiological tool TEM is limited in efficacy requiring cells to be fixed and stained although it still offers the gold standard in spatial resolution. MβCD is extremely efficacious in removing caveolar domains (as confirmed by TEM studies) but also results in the non-specific depletion of plasma membrane cholesterols. Thus non-specific effects may arise and data obtained from MβCD-based studies should be treated with some caution. Caveolae also represent mechanosensitive regions of the cell, in cardiomyocytes they act as reservoirs of membrane allowing the cell to increase its surface area in response to osmotic or mechanical stress. In the case of both stresses caveolae are observed to disappear in TEM studies (Kohl et al., 2003; Kozera et al., 2009).

Physiological investigations have revealed that caveolar depletion results in the loss of compartmentation of cAMP signaling following β_2_AR stimulation, thereby altering its effects on cardiomyocytes function. This appears to be due to the removal of protein phosphatase (PP) activity suggesting a role for caveolar localization in controlling the β_2_AR’s signaling characteristics (MacDougall et al., 2012; Wright et al., 2014). Interestingly, β_2_AR is pleiotropic and may signal via G_*s*_, G_*i*_, or β-arrestins. Removal of caveolar localization alters this capacity; caveolar localization appears to be necessary for β_2_AR to bind Gi (Xiang et al., 2002). Heart failure has been shown to significantly alter β_2_AR-cAMP compartmentation as well as caveolae number and expression of caveolae scaffolding molecules (Nikolaev et al., 2010; Feiner et al., 2011). Given the important role of the β_2_AR as a cardio-protective molecule this situation may exacerbate heart failure. The opioid receptor μOR localizes to lipid rafts in various cell types, including cardiomyocytes where it specifically localizes in caveolae. Its chronic activation leads to receptor internalization and can directly influence cAMP levels by “super-activating” AC. Methyl-β-cyclodextrin disruption of caveolae completely abolishes this “super-activation” (Zhao et al., 2006). Equally, transforming growth factor-β (TGF-β) receptors TβRI and TβRII are assumed to sit inside caveolae. There they regulate the endothelial nitric oxide synthase (eNOS; Schwartz et al., 2005) and additional TGF-β signaling downstream effectors which play a role in various physiological processes such as cell apoptosis and proliferation (Razani et al., 2001). The activation of TβRI and TβRII does not change cAMP level; at the same time otherwise increased cAMP can suppress the TGF-β-dependent signaling pathways (Schiller et al., 2010). Another receptor assumed to be situated inside the caveolae is the bradykinin type 2 receptor (Haasemann et al., 1998; Calizo and Scarlata, 2012). Activation of this receptor subtype has been shown, at least in vascular smooth muscle cells, to increase cAMP level (Webb et al., 2010). In contrast, in adult rat cardiomyocytes bradykinin type receptors appear to activate their downstream effectors without raising cAMP level, and this leads to dephosphorylation of the proteins PLB and troponin I, which reduces cardiomyocyte contractility (Ke et al., 2010). Though α_1_ adrenergic receptors (α_1_AR) appear to have no effect on cAMP levels (Bogoyevitch et al., 1993), they can elicit increased contractility and are thought to interact with the βAR signaling pathway (Brodde and Michel, 1999). Signaling of the α_1_AR, i.e., via specialized pools of phosphatidylinositol (4, 5) bisphosphate (PIP2) is localized to caveolae together with G_α*q*_ and PLCβ1 (Morris et al., 2006). It is not clear if the α_1_AR themselves are actually inside the caveolae. Instead they might be localized exclusively at the nuclear membrane. However, their downstream targets, extracellular signal-regulated kinases (ERKs) and protein kinase C (PKC) are seen to be located in caveolae (Petrashevskaya et al., 2004; Wright et al., 2008). It is generally hypothesized that caveolar localization of receptors within caveolae leads to their control by compartmentalizing these molecules with effectors which serve to inhibit their activity (Head et al., 2005). This control can be exerted by molecules which act directly upon the receptor, molecules which serve to produce (Head et al., 2006) cAMP responses or control downstream effectors of GPCR signaling, such as phosphatases (MacDougall et al., 2012).

#### T-tubules

The T-tubules are specialized domains within muscle cell types which allow efficient transmission of action potentials into the cell interior. They can be thought of as a further specialization of the plasma membrane, in a similar fashion to caveolae, as they represent modified membrane domains with specific scaffolding proteins (Louch et al., 2010). These include T-cap and Bin-1 as well as an appreciable amount of caveolin, although the presence of true caveolae in these structures remains controversial, in cardiac tissue (Wong et al., 2013). As a result, T-tubules serve as scaffolds to assemble components of ion channels and receptor cascades to exert tight control ionic fluxes in response to the respective extracellular stimuli (Bers, 2002). Certain GPCRs are found within the T-tubular regions, which appear to exert a degree of control over their signaling properties. The T-tubules of cardiomyocytes have been shown to be important organizing factors for βAR signaling and the disruption of T-tubules during pathologies alters the physiological outcome of βAR signaling (Nikolaev et al., 2010). In the adult myocardium the disruption of the T-tubular system is germane in situations of pathology (Louch et al., 2010). The general mechanism suggested for t-tubular control of GPCR signaling is compartmentation with molecular inhibitors/effectors, much like the situation in caveolae. However, the large structural aspect of T-tubules, especially those found in cardiomyocytes means a more physical role is also mooted. In our study it appeared that the T-tubular domains were able to cause a tight coupling between membrane domains rich in AKAP and PKA. The disruption of these domains leads to a β_2_AR-cAMP response which was no longer spatially localized. We suggest this alteration may lead to an altered panel of effectors for β_2_AR within diseased cardiomyocytes.

## Techniques to Study Localized cAMP Pharmacology

As the first part of the review has described, overall alterations to cellular and organ physiology by biochemical stimuli, mediated by GPCRs, are increasingly understood to be the product of many structurally defined signaling events where GPCRs, their secondary messengers and downstream effectors are tightly regulated at the sub-cellular level. Therefore to understand the true nature of a given pathway or the intrinsic efficacy of a ligand, researchers must use techniques with sub-cellular resolution.

**FIGURE 2:**
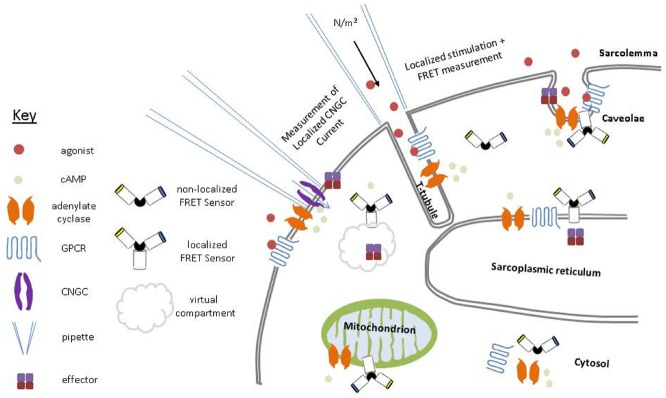
**Schematic representation of the extant modalities for measuring localized cAMP function within cells.** The investigation of cAMP signaling by patch-clamp electrophysiology, combination scanning ion conductance microscopy and Förster resonance energy transfer (SICM/FRET) and FRET microscopy utilizing sensors with subcellular localization motifs.

Historically, researchers have not been able to determine the localized pharmacology of GPCRs and instead have relied on physiological or pharmacological studies of isolated cells or tissues. Given the current state of knowledge it seems that only by studying the outcomes at the level of signaling structures/domains can we move forward in our understanding of these events. We need to know how the GPCRs, which modify cellular function, are themselves guided by micro-domains in specific cell types and how this regulation is altered by pathologies. In cardiac physiology it remains unclear whether increased aberrant signaling by GPCRs in pathologies and derangements of T-tubules are an initiator of, or a response to, abnormal cellular function. The poor spatial and temporal resolution of traditional biochemical techniques was what initially led Buxton and Brunton (1983) to question how it could be that cAMP could cause opposing biological effects. It has become evident that the localized nature of GPCR physiology demands localized measurements of secondary messengers. However, studies of the localized physiology of signaling events produced by GPCRs in domains of a radius smaller than 500 nm are rare, as a consequence of the diffraction limit of light, curtailing the ability of light microscopy methods to operate at such small scales. In recent years new methods have emerged which deal with the limitations described above. The next section will describe the current state of the art in techniques to study the localized physiology/pharmacology of GPCRs. (See Figure 2).

### Patch Clamp

The patch clamp technique was the first method to effectively study localized cellular physiology and localized receptor pharmacology (Auerbach and Sachs, 1984). This technique exploits the phenomena of ion exchange between the intra and extracellular regions of impermeable plasma membrane, via voltage sensitive and ion-selective channels. This technique has a special utility in the study of electronically excitable cells such as neurons and muscle cells (myocytes). By placing a glass micropipette, machined to provide a pore with a radius of less than a micron onto the surface of a cell and generating a high resistance seal, the holding voltage of the membrane can be set. This allows the current and therefore the activity of ion channels to be recorded. A number of studies have used a modified patch clamp technique to indirectly investigate GPCR dependent cAMP production (Rochais et al., 2004; Abi-Gerges et al., 2009; Ghigo et al., 2012). The focus of many of these studies was the adenylyl cyclase family. Most utilized the properties of certain Ca^2+^-transporting channels which are modulated by cyclic nucleotides (CNGCs) and which co-localize with adenylyl cyclases in cell membranes (Finn et al., 1996). Although the wild-type channels do not differentiate very effectively between cAMP and cGMP, mutations causing increased selectivity have been produced. Site-directed mutagenesis has been used to alter a single glutamic acid residue to a methionine (E583M) and a further compound mutation of a cysteine to a tryptophan (C460W/E583M) (Rochais et al., 2004). This produced two separate cAMP sensing channels; these have been expressed in various primary cell types (Rich et al., 2001). Consecutive patching reveals channels which give responses on the basis of local cAMP levels. The modulation of CNGCs current as a result of the application of receptor agonists or external stimuli can be obtained by standard electrophysiological means. Further work looked at the hyperpolarizing cation channels (HCN) which are between 10 and 1000 times more sensitive to cAMP than CNGCs (Rich et al., 2001). These channels are related in that they both contain an evolutionarily conserved cyclic nucleotide binding cassette domain (CNBD). Their development built upon the work of Trivedi and Kramer (1998) who pioneered what was the first method to give truly localized real-time measurement of GPCR activity, “patch-cramming.” The patch-cramming technique is based upon the activity cyclic nucleotide sensitive ion channels and involves excision of patches from the lipid membrane bi-layer of cell types which express a high density of CNGCs, for example of oocytes or retinal rod cells, with a patch clamp pipette. Through this operation an inside-out patch of cell membrane will be removed and the CNGCs within can be used to sense external cAMP levels. Calibration of these isolated channels is possible with external solutions containing predefined concentrations of cyclic nucleotides. The CNGC based pipette sensor can then be “crammed” into a cell of interest for which the level of the secondary messenger is to be determined.

### Localized Förster Resonance Energy Transfer (FRET) Sensors

A further way to study the localized pharmacology of GPCRs would be to use FRET sensors which are localized to a sub-cellular region of interest. The general approach of this technique is to produce genetically encoded peptide constructs comprising a targeting motif, a signaling molecule binding region and a fluorescent sensor component. The sensor component is usually two fluorophores which are designed, so as to be held in a specific conformation upon the folding of the peptide. One fluorophore acts as an energy donor and the other is an energy acceptor. These fluorophores are either brought into or moved out of proximity upon the binding of the messenger molecule of interest, the result is an alteration in the fluorescence of the donor fluorophore due to the phenomenon of Förster resonance energy transfer. These alterations can be measured by either ratiometric or fluorescence life-time protocols to give an indication of the relative concentration of the secondary messenger molecule of interest in the proximity of the sensor (Sprenger and Nikolaev, 2013). The location targeting motif ensures that the measurement of FRET responses after secondary, messenger activity occurs within a specific locality.

FRET-based investigation of secondary messenger activity is a somewhat immature field, but localized FRET sensor technology has been a critical component of this field since its inception. The seminal studies of Zaccolo and collaborators describe the production of sensors based upon the regulatory I and II (RI/RII) regions of PKA (Zaccolo and Pozzan, 2002). This resulted in sensors which were localized to either the PKA_RI or RII regions. These are regions which, within the cardiomyocytes, control different aspects of cellular physiology. Thereby it was made possible to demonstrate that cAMP pools activated upon Isoprenaline (βAR) or Prostacyclin (EPR) were indeed discrete (Di Benedetto et al., 2008). Interestingly, non-localized sensors were developed later than localized sensors as a solution to the problem of the high sensitivity of PKA-based constructs. As cAMP is often present at high concentrations within cells the initial class of FRET sensors were easily saturated, meaning experiments lost resolution at physiological levels of cAMP production. Non-localized sensors have most often been based upon the cAMP binding domains of EPAC1 or EPAC2 (Nikolaev et al., 2004). These sensors have shown efficacy in studies assessing the diffusion of cAMP throughout the cell (Nikolaev et al., 2006). Transgenic technology has allowed the HCN2 and Epac1 sensors to be incorporated into mouse DNA, creating strains of animal which express these sensors within every cell of their body (Nikolaev et al., 2006; Calebiro et al., 2009). cGMP sensors have been created by using the cAMP binding domains of phosphodiesterases or PKG as their detector region. There is a transgenic mouse strain with the RED DE5 cAMP sensor (Sprenger et al., 2015).

On the basis of these general sensors with lower sensitivity, localized sensors have been created by fusing various localization domains. Many if not all of the applications of these localized sensors have been focused upon investigating the molecular actors involved in modulating excitation-contraction coupling within the cardiomyocyte. This is the result of a relatively small number of groups being involved in this process and their general interest in the cardiac field. Fusions of the PKA-RI and RII regulatory domains with the non-localized Epac-1 sensor were introduced as an update of the original genetically encoded FRET sensors by the Zaccolo group and measure cAMP in PKA microdomains (Di Benedetto et al., 2008). Fusions of cGMP and cAMP sensing domains have been made with the N-termini of phosphodiesterases to investigate the dynamics of secondary messengers within the vicinity of the molecules charged with controlling their levels (Herget et al., 2008). Fascinating studies have been conducted looking at specific membrane microdomains such as the sarcoplasmic reticulum, which are beyond the reach of the pipette based approaches discussed in this review. These have investigated cAMP levels and the activity of PKA in these regions (Dyachok et al., 2006; Liu et al., 2011). Equally, the plasma membrane itself has been investigated with a fusion peptide targeted toward caveolar membranes by fusion with a motif from Lyn kinase (Wachten et al., 2010; Mohamed et al., 2011). As well as this the domains of specific adenyl cyclases have been probed by fusions of Epac2 and AC8. The membranes of mitochondria and the nucleus have been probed by fusion of mitochondrial sequences and nuclear targeting motifs to the ICUE (indicator of cAMP using Epac) class of FRET sensors (DiPilato et al., 2004; Sample et al., 2012). This area and other biophysical techniques for cyclic nucleotide measurement have been reviewed in exhaustive detail by Sprenger and Nikolaev (2013).

### Scanning Ion Conductance Microscopy/FRET (SICM/FRET)

Scanning ion conductance microscopy (SICM) was developed by Paul Hansma who realized that ion fluxes, present when performing patch clamp experiments, could also be used to image cellular topography (Hansma et al., 1989). The glass capillary pipettes of a type similar to that is utilized in patch clamping, but of higher resistance, are able to function as a scanning probe. The ion flux present between the negative electrode inside the pipette and the positive one in the bath is reduced when the pipette tip is moved in close proximity to cellular structures. This results in a drop in conductance and a measurable drop in current. By scanning the pipette across the surface of interest a 3D map of the relative variance in conductance can be built up. The system is run in a feedback mode meaning that conductance may not drop beneath a pre-defined value; this prevents the pipette from coming into contact with the sample surface. The topographical images acquired by this imaging modality give sub-optical resolution, which is defined and only limited by the radius of the pipette tip. SICM scans bear a striking resemblance to scanning electron micrograph images only cells and other biological objects are live and non-prepared, unlike the aforementioned imaging modality (Miragoli et al., 2011). Novak et al pioneered the “Hopping Mode” approach which removed image artifacts caused by large structures obstructing the scanning pipette in the x/y directions (Novak et al., 2009).

Clearly the pipette itself cannot monitor GPCRs function; this imaging modality must be multiplexed with other techniques. In our group SICM has been used in combination with FRET microscopy to study GPCR function. SICM is able to resolve complex membrane topography and the nanopipette allows application of picolitres of solution to cells. FRET microscopy can then offer a “read-out” upon the relative presence of a secondary messenger response (Nikolaev et al., 2010; Wright et al., 2014). The resolution of SICM utilized in the two studies using this hybrid technique has been around 200 nm, allowing T-tubule openings to be observed in adult cardiomyocytes. This means that the openings of individual T-tubular regions can be targeted for agonist application. The presence of cAMP responses can then be investigated at points both near and far from the region of application to assess the relative diffusion/ propagation of the cAMP response upon agonist application. The measurement of the propagation of cAMP within the cell cytosol is only possible with a non-localized FRET sensor. This makes this class of sensor important for this modality. In one of the extant studies a modified high-resolution SICM setup was able to resolve structures on the scale of 50 nm which may be caveolae, meaning that these structures could be targeted at some point in the future.

In normal cardiomyocytes transfected with a cAMP-sensing FRET construct SICM/FRET has demonstrated that β_2_AR are strictly localized to T-tubules (Nikolaev et al., 2010). Application of Isoprenaline from the nanopipette into T-tubules, but not to the areas between T-tubules (cellular sarcolemma crests) gives rise to stringently localized sub-cellular cAMP responses. This situation is subverted in failing cardiomyocytes, T-tubules are disrupted and the application of Isoprenaline to cellular crests begins to elicit β_2_AR-cAMP responses. The cyclic AMP response following β_2_AR stimulation also loses its stringent localization in space. The pathological consequences of this alteration are not clear, but it may contribute to the loss of contractile function observed in the failing myocardium and the apparent desensitization of the myocardium to sympathetic input. If this is the case then it is clear that a disruption of the sub-cellular environment in the setting of pathology, even if not modifying the intrinsic properties of receptors, can modify its extrinsic pharmacological function. A follow up study assessed the treatment of rats with an experimental gene therapy technique (Lyon et al., 2012), which caused the overexpression of the Ca^2+^ pump SERCA2a in failing cardiomyocytes. This treatment restored the T-tubular structure of the failing cardiomyocytes and the β_2_AR response was observed in the T-tubule whilst a response which was inducible at the crest was no longer present. This demonstrated the importance of maintaining discrete sub-cellular structures to enable the proper control of cAMP compartmentation.

In parallel to the disruption of T-tubular structures the caveolar domains also appeared to heavily modify β_2_AR-cAMP compartmentation (Wright et al., 2014). MβCD caused the β_2_AR-cAMP response to appear upon cellular sarcolemmal crests and to propagate throughout the cell. Further to that, the importance of cAMP compartmentation by caveolar structures was exposed by knocking down Caveolin-3 (cav-3) (Wright et al., 2014). This displacement caused the β_2_AR-cAMP response to remain localized to T-tubular domains but caused the cAMP response to propagate throughout the cell. Over-expression of cav-3 in failing cardiomyocytes was able to restore localized β_2_AR-cAMP response which was previously deranged. A novel computer model has been produced which accurately predicted the displacement of cav-3 to be more difficult at the sarcolemma in relation to the crest due to the differences in the formation of caveolae in the different regions of the cardiomyocytes (Wright et al., 2014). The latter prediction was confirmed by an entirely separate work using super-resolution confocal microscopy techniques (Wong et al., 2013).

## Conclusion

As this review demonstrates the function of GPCRs and the control of cAMP and other secondary messengers cannot be divorced from the membrane environment that these molecules are localized within. As a result only through understanding how these domains affect the function of GPCRs and control cAMP responses can one begin to understand how to rationally manipulate intracellular cAMP responses to provide benefit within the contexts of human pathology. The studies reviewed have exclusively been performed in animal models and many, as discussed, have been performed by groups with a special interest in the cardiac field. The cardiomyocyte is a singular cell type and presents a degree of structural complexity second perhaps only to that of neurons. As a result the apparent stringency of secondary messenger compartmentation by structural means may not be as essential in other cell types.

The techniques above present avenues toward the assessment of GPCR function at the level of membrane localization. The techniques are not prohibitively sophisticated, with SICM being, at least in theory, within reach of laboratories utilizing patch-clamp technology. Equally, simple ratiometric FRET is a straightforward microscopy technique and the combination of SICM/FRET requires only the co-ordination of both techniques. Adenoviral and plasmid constructs encoding various localized and general FRET sensors targeted at different second messengers are becoming widely available to the research community. The emergence of a greater number of manufacturers on the market for this instrumentation will drive more researchers to adopt what has proven to be very powerful experimental approaches.

### Conflict of Interest Statement

The authors declare that the research was conducted in the absence of any commercial or financial relationships that could be construed as a potential conflict of interest.
